# Postsynaptic VAMP/Synaptobrevin Facilitates Differential Vesicle Trafficking of GluA1 and GluA2 AMPA Receptor Subunits

**DOI:** 10.1371/journal.pone.0140868

**Published:** 2015-10-21

**Authors:** Suleman Hussain, Svend Davanger

**Affiliations:** Laboratory for Synaptic Plasticity, Division of Anatomy, Department of Molecular Medicine, Institute of Basic Medical Science, University of Oslo, P.O. Box 1105 Blindern, 0317 Oslo, Norway; University of Leicester, UNITED KINGDOM

## Abstract

Vertebrate organisms adapt to a continuously changing environment by regulating the strength of synaptic connections between brain cells. Excitatory synapses are believed to increase their strength by vesicular insertion of transmitter glutamate receptors into the postsynaptic plasma membrane. These vesicles, however, have never been demonstrated or characterized. For the first time, we show the presence of small vesicles in postsynaptic spines, often closely adjacent to the plasma membrane and PSD (postsynaptic density). We demonstrate that they harbor vesicle-associated membrane protein 2 (VAMP2/synaptobrevin-2) and glutamate receptor subunit 1 (GluA1). Disrupting VAMP2 by tetanus toxin treatment reduces the concentration of GluA1 in the postsynaptic plasma membrane. GluA1/VAMP2-containing vesicles, but not GluA2/VAMP2-vesicles, are concentrated in postsynaptic spines relative to dendrites. Our results indicate that small postsynaptic vesicles containing GluA1 are inserted directly into the spine plasma membrane through a VAMP2-dependent mechanism.

## Introduction

Synapses are junctions between neurons where the flow of information in the brain can be modified [1]. The most widely used excitatory neurotransmitter is the amino acid glutamate [2]. Glutamate receptors of the AMPA (α-Amino-3-hydroxy-5-methyl-4-isoxazolepropionic acid) class are tetramers of different subunits (GluA1-4) [3].

Synaptic plasticity, through changes in the postsynaptic plasma membrane concentration of the AMPA receptors, enables the organism to adapt to changes in the environment [4, 5]. The receptors, or their subunits, recycle between cytoplasmic and membrane pools [6]. This cycling may allow fast, regulated changes in synaptic AMPA receptor concentration, thus enabling changes in synaptic strength [7]. Indirect evidence indicates a vesicular mechanism for this recycling [8]. To our knowledge, no previous investigations have directly demonstrated the presence of such receptor-containing postsynaptic vesicles.

One of the last steps in the transport of glutamate receptors to the synapse is their delivery into the specialized dendritic membrane of the spine postsynaptic density (PSD). The exocytosis of receptors is required for long-term potentiation (LTP) [9–11], in addition to the constitutive insertion of new receptors in basal conditions [9]. Receptors can be either directly inserted into the synapse, or into the extra-synaptic membrane, followed by their lateral diffusion and subsequent trapping at synaptic sites. Regulated insertion of AMPA receptors may be initiated by NMDA (N-methyl-D-aspartate) receptor activation [12]. Though receptors are probably assembled prior to their transport to the synapses, we do not know whether the receptors may also be modified locally by single subunit trafficking to the postsynaptic plasma membrane for assembly there.

AMPA receptors are most likely synthesized as monomers in the endoplasmic reticulum, before subsequent insertion into the endoplasmic reticular membrane. Here they assemble differentially into dimers of dimers, i.e. tetramers [13, 14]. Tetrameric AMPA receptors then continue to the Golgi apparatus and exit the trans-Golgi network with trafficking vesicles. Some investigations, however, point to the possibility of differential trafficking of GluA1- and GluA2-containing receptors [15, 16]. GluA1 and GluA2 subunits can also be synthesized in dendrites in an activity-dependent or an activity-independent manner [17].

Exocytosis in neurons requires proteins known as Soluble NSF Attachment Protein Receptors (SNAREs), membrane proteins that are involved in many intracellular fusion events. According to the SNARE hypothesis, membrane fusion results from the interaction of specific vesicle and target SNAREs that bring their respective membranes into close opposition leading to fusion [18]. An important step in these processes is the assembly of a complex consisting of a small number of proteins, forming the core SNARE complex. In nerve terminals, this complex consists of VAMP2/synaptobrevin-2, which resides at presynaptic vesicle membranes, and syntaxin-1 and SNAP-25 at the corresponding presynaptic plasma membrane [19].

In addition to their crucial role in presynaptic exocytosis [19–22], SNARE proteins are main candidates for a regulatory role in the fusion of receptor-containing organelles with the postsynaptic plasma membrane [10, 23–26]. VAMP is a small integral membrane protein of synaptic vesicles in vertebrates and invertebrates. The protein is highly conserved across evolution. VAMP1 and VAMP2 are brain-specific and expressed in a non-overlapping pattern, though VAMP2 is much more ubiquitous then VAMP1 in the CNS [27].

We wanted to determine whether the vesicle SNARE VAMP2 is present in postsynaptic spines in the brain, whether it is associated with postsynaptic vesicles containing AMPA receptor subunits, and if it contributes to the exocytotic insertion of these AMPA receptor subunits into the plasma membrane.

## Material and Methods

The crucial technology that facilitated these observations was immunogold postembedding electron microscopy with antibodies against glutaraldehyde-fixed antigen [28], in combination with freeze-substituted brain tissue fixed with formaldehyde and very low concentrations of glutaraldehyde, without osmium treatment [29]. The freeze-substitution technique has proven effective in visualizing synaptic-like microvesicles in other sites than presynaptic terminals [30, 31].

### Antibodies

Anti-VAMP2 was raised in rabbit immunized with recombinant VAMP2/synaptobrevin [32] protein (amino acid 1–96, 15% identical with VAMP1 amino acid 1–96) fixed in 1.25% glutaraldehyde and mixed with Freund’s adjuvant. The VAMP2 construct (pGEX-KG vector) was a generous gift from Richard Scheller. Crude antiserum was affinity-purified with recombinant VAMP2 protein (affi-gel column). Anti-VAMP2 was used at 1:10,000–50,000 for western blotting, 1:10–1:50 for postembedding electron microscopy (EM), 1:100 for immunofluorescence confocal microscopy and 5–10 μg/50 μl beads for immunoprecipitation. Anti-GluA1 (GluR1) (Alomone Labs, Jerusalem, Israel, Cat#AGC-004) was used at 1:500 for immunofluorescence. Anti-GluA1 (GluR1) (Millipore, MA, USA, Cat#AB1504) was used at 1:20 for electron microscopy and at 5–10 μg/50 μl beads for immunoprecipitation. Anti-GluA1 (GluR1) (Upstate, NY, USA, Cat#07–660) was used at 1:1000 for western blotting. Anti-GluA2 (GluR2) (Alomone Labs, Jerusalem, Israel, Cat#AGC-005) was used at 1:500 for immunofluorescence, 1:25 for electron microscopy, 1:1000 for WB and 5–10 μg/50 μl beads for immunoprecipitation. Anti-synaptophysin (Millipore, MA, USA, Cat#MAB368) was used at 1:50,000 for western blotting and at 1:200 for immunofluoroscence. Anti-PSD95 (Novus Biological, CO, USA, Cat#NB300-556) was used at 1:75 for immunofluorescence. Anti-phosphate-activated glutaminase [33] (in-house) was used at 1:150,000 for western blotting. Anti-ß-tubulin (Tuj1) (Covance, CA, USA, MMS-435P) was used at 1:100 for immunofluorescence and 1:10,000 for western blotting. Secondary antibodies: Donkey anti-rabbit Cy3 (Jackson Immuno, MD, USA, Cat#711-165-152) and donkey anti-mouse A488 (Invitrogen, CA, USA, Cat#A21202) was used at 1:1000 for immunofluorescence. Goat anti-rabbit alkaline phosphatase (Sigma, MO, USA, A3687) and Goat anti-mouse alkaline phosphatase (Sigma, MO, USA, Cat#A3562) was used at 1:10,000 for western blotting. IgG coupled to 10 or 20 nm colloidal gold (Abcam, Cambridge, UK, Cat#ab27234) and (British BioCell International, Cardiff, UK, Cat#R14007) was used at 1:20.

### Animals

All animal experimentation was carried out in accordance with the European Communities Council Directive of 24 November 1986 (86/609/EEC). Formal approval to conduct the experiments described was obtained from the animal subjects review board of the Norwegian Governmental Institute of Public Health (Oslo, Norway). Care was taken to minimize the number of animals used and to avoid suffering. According to established routines in our EM lab, adult male Wistar rats, 2–4 months, (from Scanbur, Nittedal, Norway), weighing 250–300 g, were used for EM, and 1–4 day-old Wistar rats for primary hippocampal cultures. Similarly, adult PVG male rats, 2–4 months, (Scanbur), weighing 200–250 g, were used for western blotting. VAMP2 knock out mice brains [34] were generously provided by Ege Kavalali, University of Texas Southwestern Medical Center, Dallas, TX 75390, USA.

### Immunocytochemistry

#### Perfusion fixation

For electron microscopy studies, the rats were deeply anesthetized with Equithesin (0.4 ml/100 g body weight), followed by intracardiac perfusion with 10–15 s flush of 4% Dextran-T70 in 0.1 M sodium phosphate buffer (pH 7.4), before perfusion with a mixture of 4% formaldehyde and 0.1% glutaraldehyde in the same buffer. Time for perfusion was approximately 15 minutes. The rats were then left overnight ON in a cold room. The next day, the brains were carefully dissected out and stored in a sodium phosphate buffer (0,1 M with 0.4% FA and 0,01% GA).

#### Electron microscopy

Small (0.5–1.0 mm) blocks dissected out from the CA1 area of the hippocampus were freeze-substituted, sectioned, and immunolabeled essentially as described previously [29, 35]. For double labeling, the sections were first incubated with either rabbit polyclonal anti-GluA1 or anti-GluA2 followed by goat anti-rabbit coupled to 20 nm colloidal gold. The sections were exposed to formaldehyde vapor at 80 C for 1 h and thereafter incubated with rabbit polyclonal anti-VAMP2, followed by goat anti-rabbit coupled to 10 nm colloidal gold. The sections were the contrasted with uranyl acetate and lead citrate and examined with a Fei Tecnai 12 electron microscope.

#### Quantification and statistical analysis of immunogold labelling

Electron micrographs from rats (*n* = 3) were obtained from asymmetric synapses (Schaffer collateral synapses) from the middle layer of stratum radiatum of the CA1 region of the hippocampus. VAMP2 immunolabeling was quantified as the number of gold particles/μm of membrane length in asymmetric synapses and as the number of gold particles/μm^2^ in the intracellular compartment. One hundred eighty profiles from each region of interest were quantified (60 profiles from each rat). Specific plasma membrane and cytoplasmic compartments were defined and used for quantifications. The synaptic lateral membranes were defined for convenience of measurement as equal to the length of the PSD, on both sides of the PSD or AZ, for all synapses. Only synaptic profiles with clearly visible synaptic membranes and postsynaptic density were selected for quantitative analysis. An in-house extension to the analySIS software connected with SPSS (SPSS Inc, Chicago, IL, USA) was used to quantify the gold particle labeling of the regions of interest. The software calculated area gold particle density (number per unit area) over cytoplasmic compartments and linear particle density (number per unit length of curve) over membrane domains. In the latter case, it measured the distance from each particle-center to the membrane, and included only those particles which were within an operator-defined distance from the curve segment. For plasma membranes, the inclusion distance was symmetric between -21 nm, and +21 nm (negative signifying an intracellular location). Data for particles were collected in ASCII files as flat tables and exported to SPSS for further statistical and graphical analysis.

ImageJ was used to quantify double immunogold labeling. Gold particles located within a 77 nm radius from each other were classified as colocalized. The distance 77 nm was determined by summarizing the length of 2 x primary antibody (16 nm), length of 2 x secondary antibody (16 nm), radius of 10 nm gold particle (5 nm), radius of 20 nm gold particle (10 nm) and diameter of postsynaptic vesicle (30 nm).

For specificity control of VAMP2 immunogold labeling, we quantified gold particle densities over presynaptic, vesicle-filled cytoplasm in newborn knockout and wild type mouse hippocampi (after immersion fixation in our standard fixative), and in adult wild type rat hippocampi with and without preincubation of the antibody with identical VAMP2 protein to that which was used for immunization. The quantified results were analyzed with a Mann-Whitney test.

Homozygote VAMP2 knock out animals die immediately after birth [34], at a different developmental stage from the adult rat brains we used, and perfusion fixation of newborn mice is technically challenging, with a risk of altered perfusion parameters compared to adult rats. The abundant pool of presynaptic vesicles was used as our region of interest for quantification in these control experiments.

### Immunoisolation of vesicles

GluA1, GluA2 and VAMP2 antibody was coupled to Dynabeads Protein A (Dynal, Oslo, Norway), [36]. Vesicle fractions were incubated with beads overnight under rotation at 4°C. The flow-through and the beads fraction were separated, rinsed, collected and homogenized in SDS solubilization buffer (10 mg/ml SDS, 5 mM EDTA and 10 mM sodium phosphate buffer, pH 7.4). The homogenates were run on 14–20% SDS-acrylamide gels. There is no well-established protocol for distinctly separating pre- and postsynaptic vesicles in a biochemical preparation, so our samples evidently contained both types. There is no reason to believe, however, that all VAMP2-positive vesicles contain GluA1, as presynaptic vesicles (with VAMP2) would probably be low in GluA1 concentrations.

### Preparation of synaptosome and vesicle membrane fractions

Ten rats were decapitated, and the brains were dissected out, and submerged in ice-cold Hepes-buffered sucrose (0.32 M sucrose, 4 mM Hepes, pH 7.4) containing a protease inhibitor cocktail (0.1 mM PMSF, 1.5 μg/ml ea. aprotin, 10 μg/ml ea. antipain/ leupeptin, 10 μg/ml ea. chymostatin/pepstatin, 0.1 mg/ml benzamidine) and a phosphatase inhibitor cocktail (2 mM EGTA, 50 mM NaF, 10 mM sodium pyrophosphate, 20 mM -glycerophosphate, 1 mM para-nitrophenylphosphate (PNPP), 1 μM microcystin LR, 1 mM sodium orthovanadate, 0.1 mM ammonium molybdate). The tissue was homogenized in a Hepes buffer with a glass-Teflon homogenizer (900 rpm, 10–15 strokes) and centrifuged (800–1000 μ*g*, 10 min, 4°C). The postnuclear supernatant S1 was centrifuged (10,000 *g*, 15 min), and the pellet containing crude synaptosomes was resuspended in 10 volumes of Hepes-buffered sucrose and centrifuged (10,000 *g*, 15 min). The synaptosomal fraction was resuspended in 10 mM sucrose, centrifuged (161,000 *g*, 25 min); the gradient interphase was collected and diluted in Hepes-buffered sucrose, and centrifuged again (161,000 *g*, 25 min) to get a pellet containing pure synaptosomes. To extract synaptic vesicles, the pure synaptosome pellet was lysed by hypo-osmotic shock in nine volumes of ice-cold H2O plus protease/phosphatase inhibitors and three strokes with a glass-Teflon homogenizer; this solution was adjusted to 4 mM Hepes and mixed at 4°C for 30 min to ensure complete lysis. The lysate was centrifuged (161,000 *g*, 25 min), before the resulting supernatant again was centrifuged (165,000 *g* for 2 h) and then resuspended in 50 mM Hepes, 2 mM EDTA plus protease/phosphatise inhibitors.

### Immunoblotting

The homogenates were run on 4–20% SDS–acrylamide, and then electroblotted onto PVDF membrane (Hoefer Scientific Instruments, San Francisco, CA, USA) and immunostained with primary antibodies and horseradish peroxidase-linked secondary antibody (Amersham Biosciences, UK). The signal was detected by fluorescence using ECF substrate (Amersham Biosciences, UK). The fluorescence signals were visualized by a fluorescence digital camera detection system (Typhoon and Kodak scanner).

### Preparation of hippocampal neuronal cultures

Primary hippocampal cultures containing both neurons and glial cells of 1–4 day-old rats (Wistar) were prepared as previously described [37]. Briefly, cultures were prepared from 1–4 day-old rats. They were maintained in cell medium (Gibcos MEM with the addition of 30 mg/100 ml glutamine; 2.5 mg/100 ml insulin; 5–10% fetal calf serum; 2 ml/100 ml B 27 and 2–10 micro liter/100 ml ARA-C) in 5% CO_2_, 95% air incubator at 37°C. The cells were fixed in 2.5% glutaraldehyde (GA) and 1% formaldehyde (FA) by the following procedure: freshly prepared fixative (2% FA in 0.1 M sodium phosphate buffer) was heated to 37°C before adding it to the culture medium (equal volumes). After 30 min this mixture was substituted with 4% FA in 0,1 M sodium phosphate buffer ON. The cells were stored in 0,1 FA in 0,1 M sodium phosphate buffer.

### Treatment of neuron cultures with tetanus toxin and Bafilomycin A1 and confocal microscopy scanning

Cultures were incubated with 13.3 nM tetanus toxin or 1 μM Bafilomycin A1 in culture medium overnight (ON) before they were fixed. They were labeled with primary antibody in 2% (v/v) normal calf serum and 1% (w/v) bovine serum albumin and 0.4% saponin in 0.1 M sodium phosphate buffer at pH 7.4 (ON). Saponin was omitted in GluA1- or GluA2-labeling against the extracelluar epitope. The cultures were rinsed in the buffer, incubated for 30 min with secondary antibodies and rinsed again in the buffer. The cultures were mounted with fluormont mounting media (Southern Biotech), and examined with an Axioplan 2 equipped with a LSM 5 Pascal scanner head (Carl Zeiss, Heidelberg, Germany). Tetanus toxin does not cleave VAMP2 that is already assembled in a SNARE complex [38]. Thus, even after toxin treatment, there will be some insertion of GluA1 receptor subunits associated with already docked vesicles.

### Quantification and statistical analysis of hippocampal neuronal cultures

Quantification of GluA1 immunofluorescence intensity was performed with LSM image analyzer software over morphologically identified boutons in both the control and experiment groups (*n* = 1000 synapses for the tetanus group, and *n* = 500 synapses for the Bafilomycin group). Fluorescence intensities of individual boutons were analyzed in control and tetanus or Bafilomycin-treated neuronal cultures. The cultures used for quantification were from the same batch of animals, and were used on the same day. In all cultures, images were taken from the center of the culture, where the density of synapses was comparatively equal and greatest. We identified neuronal cell somata and followed the dendrites peripherally. Along these dendrites, synapses were localized as fluorescent puncta. We used fluorescence intensity as a measure of glutamate receptor concentration in these synapses. Furthermore, we quantified the staining intensity of single synapses. The results were independent of the total number of synapses in each culture. LSM image analyzer software was used to generate a histogram of the intensities along a straight line across individual boutons, and maximum immunofluorescence intensity from each bouton was obtained. Means were then calculated for the population of boutons in a given culture. Statistical analyses were performed in SPSS for Windows (SPSS Inc., Chicago, IL). The quantified results with tetanus toxin treatment were analyzed with independent samples *T*-test. The quantified results with Bafilomycin A1 treatment were analyzed with a Mann-Whitney test.

## Results

### Specificity of in-house VAMP2 antibody

**Fig 1:**
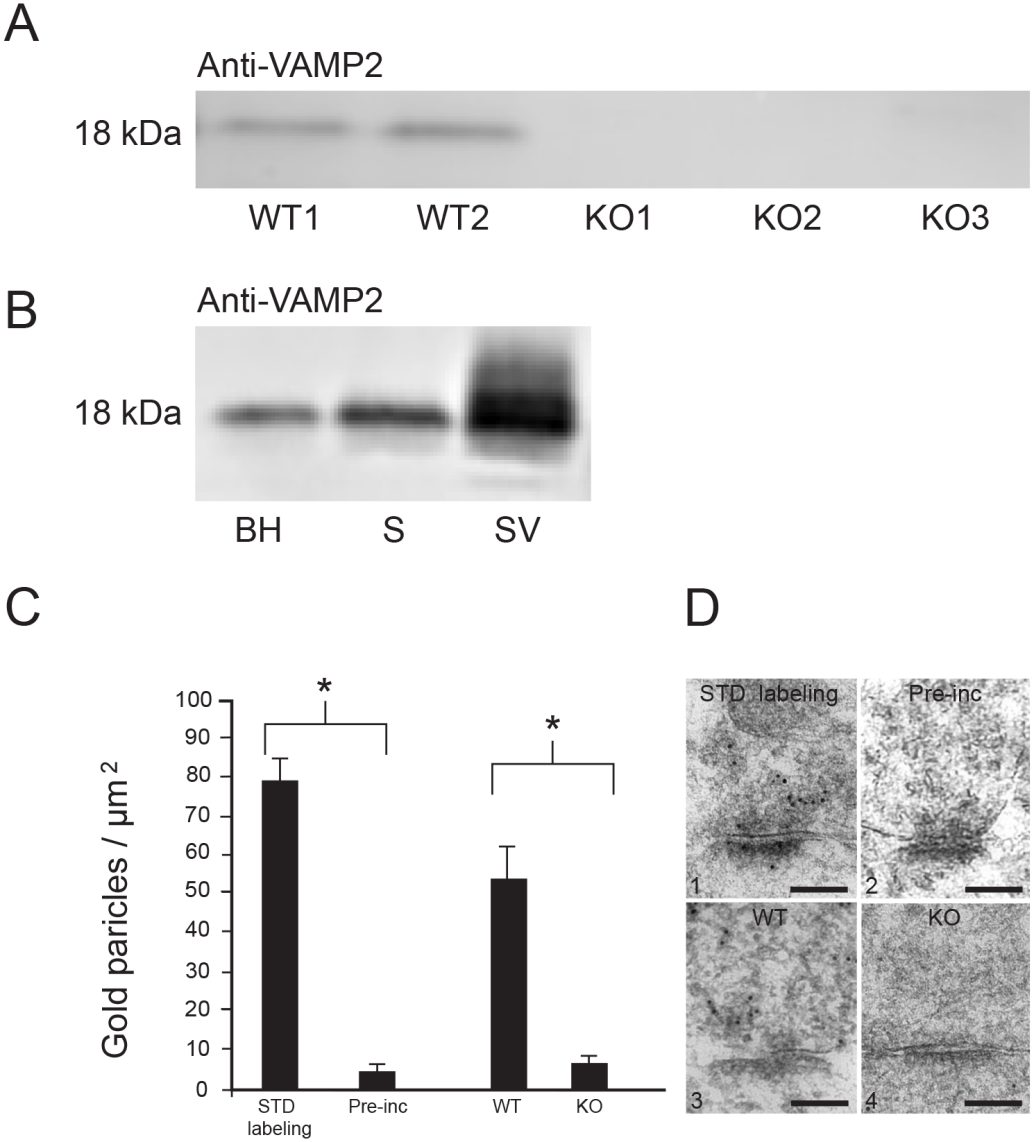
Specificity of in-house VAMP2 antibody. (*A*) Brain homogenates from wild type (WT1–2) and knockout (KO1–3) mice. The VAMP2 band at 18kDa is absent from brains of the three knockout mice. Protein loaded 30 μg. (*B*) Western blots from wild type rat brain. BH: brain homogenate; S: crude synaptosomes; SV: synaptic vesicles. Only one band was seen in each preparation. Protein loaded 10 μg. (*C*) Quantitative analysis of immunogold labeling with anti-VAMP2 of presynaptic cytoplasm at excitatory synapses in hippocampus, preincubated with VAMP2 protein. Ultrastructural quantification of VAMP2 immunogold labeling of presynaptic cytoplasm at excitatory synapses in hippocampus of VAMP2 KO mice. Statistical significance (*p* < 0.001). (D) Electron micrographs showing labeling for VAMP2. (D1) Standard labeling, i.e. normal immunogold without pre-incubation with antigen. (D2) Pre-incubation with VAMP2 antigen. (D3) Standard labeling of wildtype. (D4) Labeling of VAMP2-KO tissue. Scale bar: 125 nm.

Our anti-VAMP2 antibody was characterized at the western blot and electron microscopy levels. First, we used the antibody on western blots of brain homogenate from newborn wild type and VAMP2 knockout mice [34] (Fig 1A). Immunoblots from wild type mice show one band, as expected at 18 kDa, while this is not present with the VAMP2 knockout brain homogenate. This band was also present with rat brain homogenate, and it was further strengthened in synaptosomal and synaptic vesicle fractions, as expected (Fig 1B). In order to specifically test the antibody at the electron microscopical level, we performed immunogold labeling of hippocampus from newborn VAMP2 knockout mice (Fig 1D4) and wild type mice (Fig 1D3), immersion fixed with the same fixative as the one used for the rat experiments. Quantification of gold particle densities over vesicle-filled, presynaptic, hippocampal terminals at the electron microscopical level showed an almost eightfold higher labeling in wild type compared to knockout animals (*n* = 20 in both groups, *p* < 0.001, Mann-Whitney *U* test) (Fig 1C). We used immersion fixation of newborn VAMP2 knockout mouse hippocampus for this control, as rat VAMP2 knockout animals were not available, and the mouse knockouts die immediately after birth. We also labeled adult rat hippocampal terminals at the electron microscopical level with antibody after pre-incubation with the same antigen used for immunization of the rabbits (Fig 1D2), where control labeling of presynaptic vesicles without pre-incubation (Fig 1D1) was almost seventeen-fold higher (*n* = 20 in both groups, *p* < 0.001, Mann-Whitney *U* test) (Fig 1C). These experiments show high specificity and selectivity of our in-house VAMP2 antibody, also at the electron microscopical level.

### VAMP2 expression in the brain

**Fig 2:**
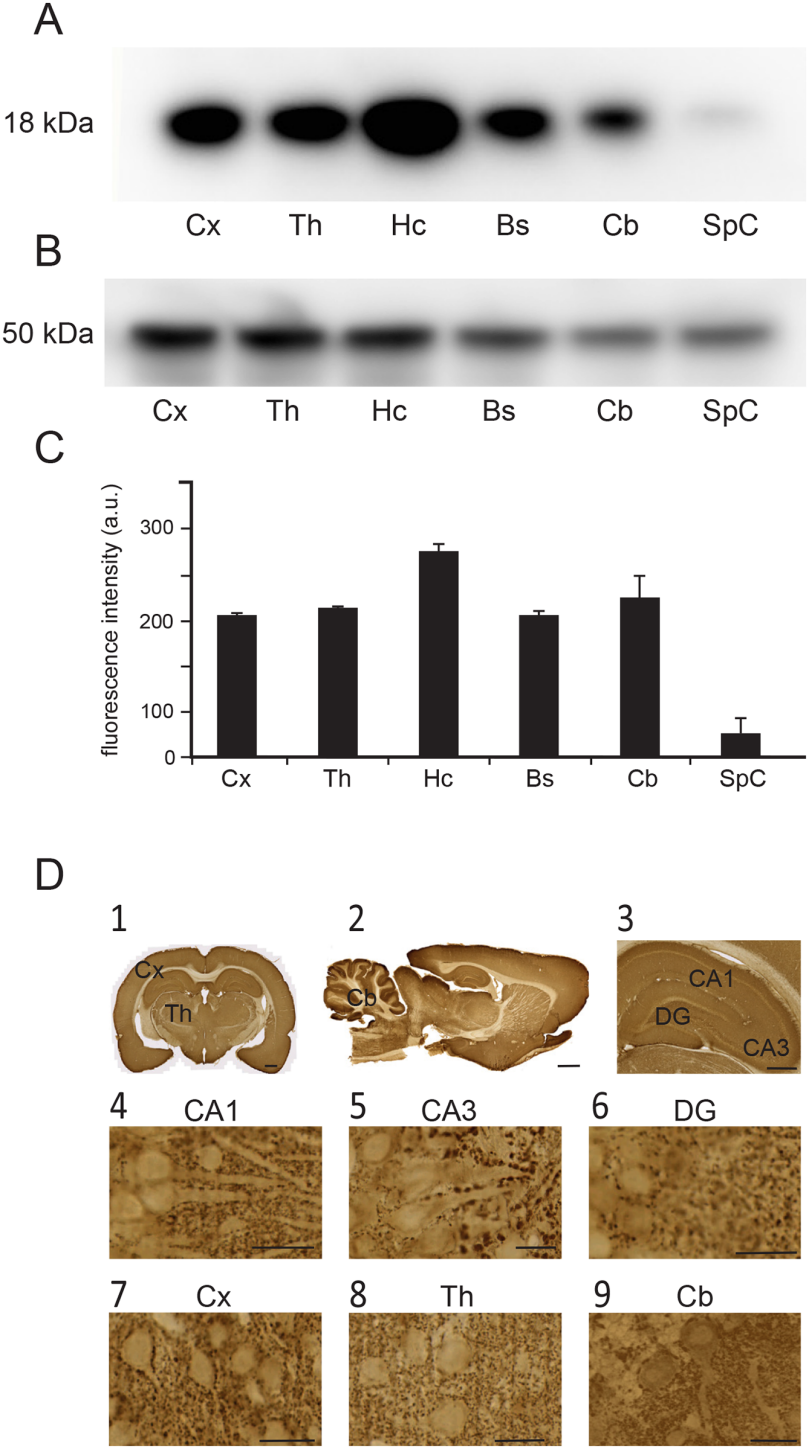
Regional VAMP2 expression in the rat brain. (A) Staining of homogenates from different brain regions with anti-VAMP2, i.e. cortex (Cx), thalamus (Th), hippocampus [64], brain stem (Bs), cerebellum (Cb), and spinal cord (SpC). Protein loaded 7,5 μg. (*B*) Staining of different brain regions with anti-beta-tubulin. Protein loaded: 5 μg. (C) Quantitation of intensities of bands seen above, indicating highest concentration in the hippocampus, lowest in the spinal cord. Fluorescence intensity of anti-VAMP2 staining was normalized to anti-beta-tubulin staining. (D) Vibratome sections immunostained for VAMP2. Anti-VAMP2 antibody produced immunoperoxidase-staining of neurons in hippocampus, cerebellum, cerebral cortex and thalamus (*D1–D3*). In the hippocampus, the somata and proximal dendrites of pyramidal neurons were strongly stained in sub-regions of CA1 (*D4*) and CA3 (*D5*), as were the granule cells in dentate gyrus (*D6*). Strong immunostaining was found in purkinje cell somata and the proximal dendrites in the cerebellar cortex (*D9*), and in the pyramidal cells of the cerebral cortex (*D7*). Immunolabeling was also observed in thalamic neurons (*D8*). Immunoperoxidase staining. Scale bar: (*D1*): 1000 μm. (*D2*): 2000 μm, (*D3*): 500 μm, (*D4–D8*): 20 μm, (*D9*): 50 μm.

To determine relative concentrations of VAMP2 in different brain regions, we performed quantitative western blot (Fig 2A–2C). Results from these experiments show that the highest concentration is found in the hippocampus, the lowest in the spinal cord (Fig 2C). The cortex, thalamus, brain stem and cerebellum showed almost equal expressions of VAMP2. Staining of different brain regions with the neuronal marker anti β-tubulin was used as the loading control for quantitative western blot experiments (Fig 2B). Light microscopy revealed strong immunostaining of VAMP2 in the cerebral cortex, hippocampus, thalamus and cerebellum (Fig 2D1–2D3), similar to what has been shown with mRNA hybridization [27]. At higher magnification, somata and proximal dendrites of principal neurons were weakly to moderately immunopositive for VAMP2, but were bordered with characteristic punctate staining, as would be expected from synapses (Fig 2D4–2D9). The results were in line with published anti-VAMP2 immunostaining.

### Ultrastructural localization of VAMP2

**Fig 3:**
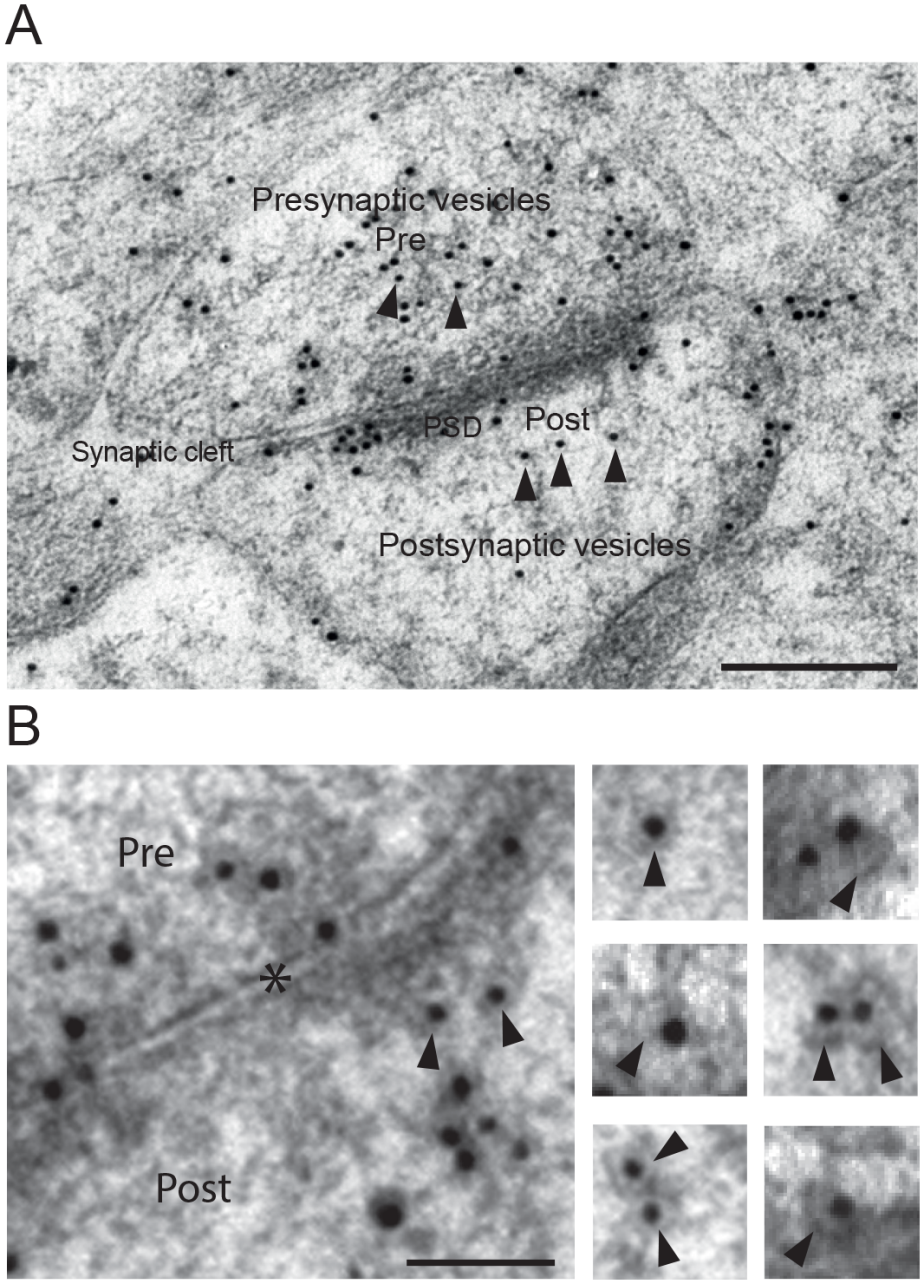
Electron micrographs showing VAMP2 immunogold labeling of vesicles in asymmetric synapses from the CA1 region of the rat hippocampus. (*A*) Gold particles are present over pre- and postsynaptic vesicles (arrowheads), as well as some at synaptic plasma membranes. Scale bar: 200 nm. (*B*) Selected postsynaptic vesicles (arrowheads) in higher magnification. Presynaptic vesicles are also seen. Synaptic cleft is marked with an asterisk. Scale bar: 75 nm.

In order to determine the localization of VAMP2 within the synapse, we analyzed postembedding immunogold labeling of the protein in Schaffer collateral synapses in ultrathin sections from the stratum radiatum of the CA1 region in rat hippocampus (Fig 3). Many presynaptic vesicles were labeled, with gold particles localized either close to the vesicle membrane, or within the vesicle lumen (Fig 3A). The latter indicated that the secondary antibody-coated gold particle was attached to more than one VAMP2 epitope along the vesicle membrane. Gold particles were also accumulated along the presynaptic active zone, probably resulting from vesicles recently fused with the plasma membrane. Confirming our hypothesis, however, labeling of postsynaptic spines was also seen. Specifically, both the PSD and scattered postsynaptic vesicles were labeled with the VAMP2 antibody (Fig 3A and 3B). These vesicles had a mean diameter of 25 nm, i.e. at the smaller end of the presynaptic vesicle range [26], which in our material had a mean diameter of 35 nm. The small postsynaptic vesicles were located deeply within the postsynaptic cytoplasm or adjacent to the plasma membrane, both at the PSD and lateral plasma membrane. Clusters of a few VAMP2 positive vesicles were often seen at the plasma membrane close to the PSD (Fig 3A). Again, gold particles were seen both within the vesicle lumen, as well as on the vesicle membrane. Electron-lucent areas were seen between centrally located gold particles in the vesicles and the vesicle membrane, showing that the 10 nm gold particles did not completely fill the space of the lumen.

**Fig 4:**
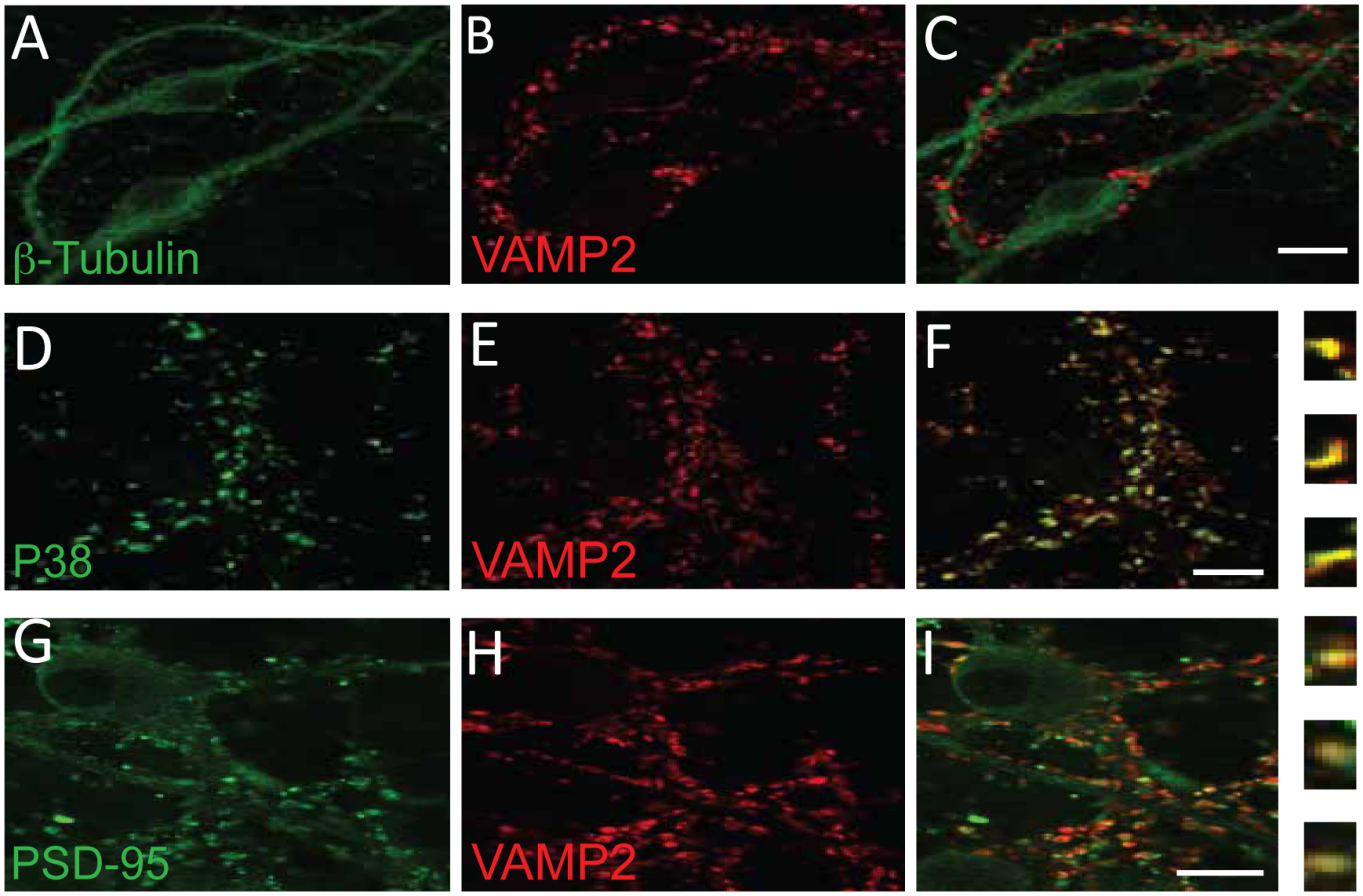
Confocal images of dissociated hippocampal cultures. Double labeling with anti-TUJ1 (*A*) and anti-VAMP2 (*B*) demonstrates that VAMP2 is located along the dendrites and gives characteristic punctate labeling (*C*). Double labeling with antibodies against synaptophysin (P38) (*D*) and VAMP2 (*E*) indicates that VAMP2 is colocalized with the presynaptic marker (*F*). Double labeling for PSD-95 (*G*) and VAMP2 (*H*) shows partial colocalization postsynaptic (*I*). Small, high-resolution pictures of single synapses from (*F*) and (*I*) are shown in the right part of these images, respectively. Scale bar: 20 μm.

In conclusion, postsynaptic spines contain organelles that may be capable of trafficking between cytoplasmic and plasma membrane compartments, i.e. vesicles carrying the SNARE protein VAMP2. To further confirm synaptic localization of VAMP2, we performed double immunofluorescence labeling of cultured hippocampal neurons with VAMP2 and synaptic markers. Immunolabeling showed punctate staining with anti-VAMP2 along neuronal dendrites (Fig 4A–4C). As expected, VAMP2 colocalized with a presynaptic vesicle marker, synaptophysin (Fig 4D–4F), and with a postsynaptic marker, PSD-95 (Fig 4G–4I).

### VAMP2 concentration in subregions of the synapse

**Fig 5:**
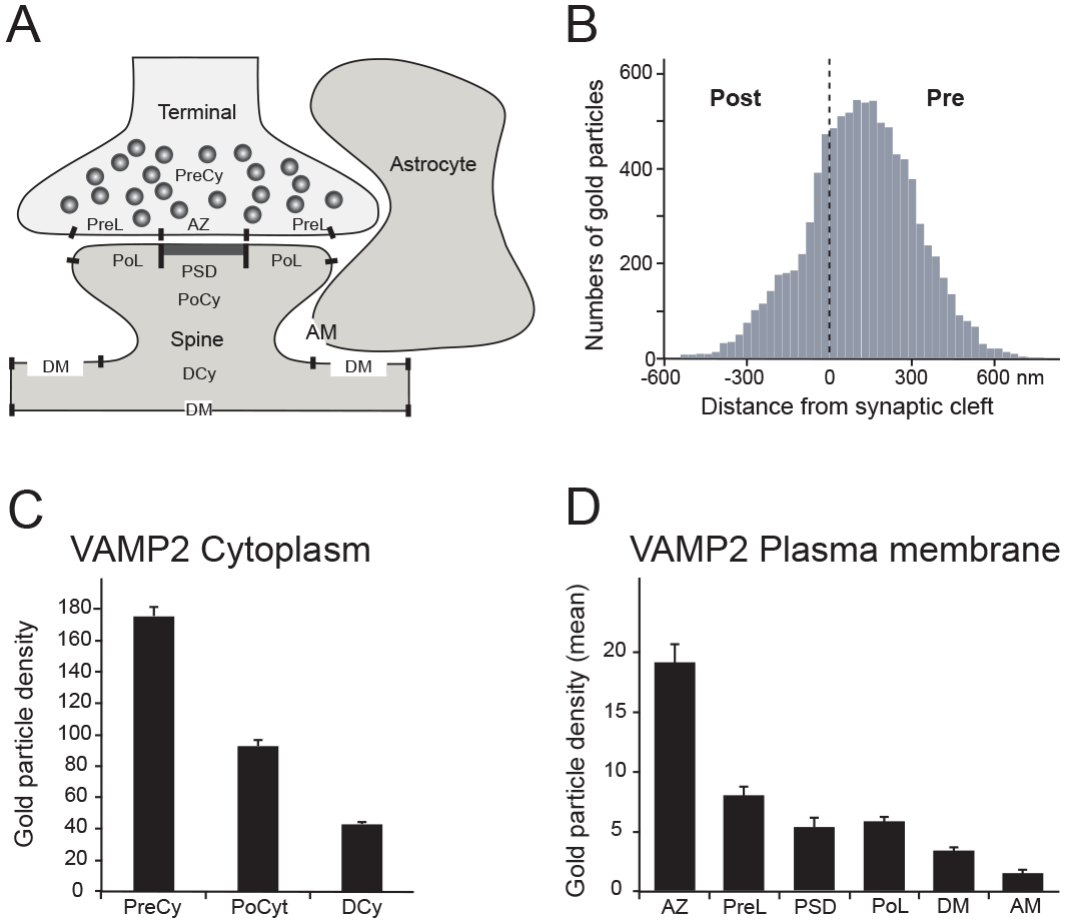
Quantitation of synaptic VAMP2 immunogold labeling. (*A*) Schematic drawing showing regions of interest in electron micrographs. PreCy: Presynaptic Cytoplasm. PreL: Presynaptic Lateral plasma membrane. AZ: Active Zone. PoCy: Postsynaptic Cytoplasm. PoL: Postsynaptic Lateral membrane. PSD: PostSynaptic Density. DCy: Dendrite Cytoplasm. DM: Dendritic plasma Membrane. AM: Astrocyte plasma Membrane. (*B*) Transverse histogram depicting the mean number of gold particles at every 30 nm distance from the center of the synaptic cleft; negative values are postsynaptic, positive values are presynaptic. The peak is about 200 nm from the center of the synaptic cleft, but significant levels are seen also in postsynaptic cytoplasm. (*C*) Mean immunogold labeling over cytoplasmic regions of interest. (*D*) Mean immunogold labeling over plasma membrane regions of interest.

In order to determine the relative concentration of VAMP2 in different subregions of the synapse, we quantified the mean number of immunogold particles over different cytoplasmic and membrane regions of interest (Fig 5A). The density of presynaptic VAMP2 peaked at about 70–140 nm from the active zone (Fig 5B), a subsynaptic region that is dense in vesicles. The postsynaptic spine cytoplasm density of VAMP2 labeling was about half that of the presynaptic cytoplasm, but still more than double the density in dendritic cytoplasm (Fig 5C). Plasma membrane labeling in the presynaptic terminal was concentrated in the active zone, with labeling more than double the density found over presynaptic lateral membrane (Fig 5D), as would be expected for a membrane domain specialized for presynaptic vesicle exocytosis. Postsynaptically, however, there was not a similar difference between synaptic and lateral synaptic plasma membrane labeling. We observed about the same concentration over the PSD as over the postsynaptic lateral membrane (Fig 5D), indicating that exocytosis may occur at both these sites. The density at the PSD-related plasma membrane, however, was markedly higher than at the dendritic plasma membrane, which again was higher than the astrocytic plasma membrane. The highest postsynaptic concentrations are clearly found at the PSD or close to it. These observations indicate that vesicle fusion with the postsynaptic spine plasma membrane may occur both directly at the synapse as well as in lateral spine areas.

### Postsynaptic colocalization of AMPA receptor subunits and VAMP2

**Fig 6:**
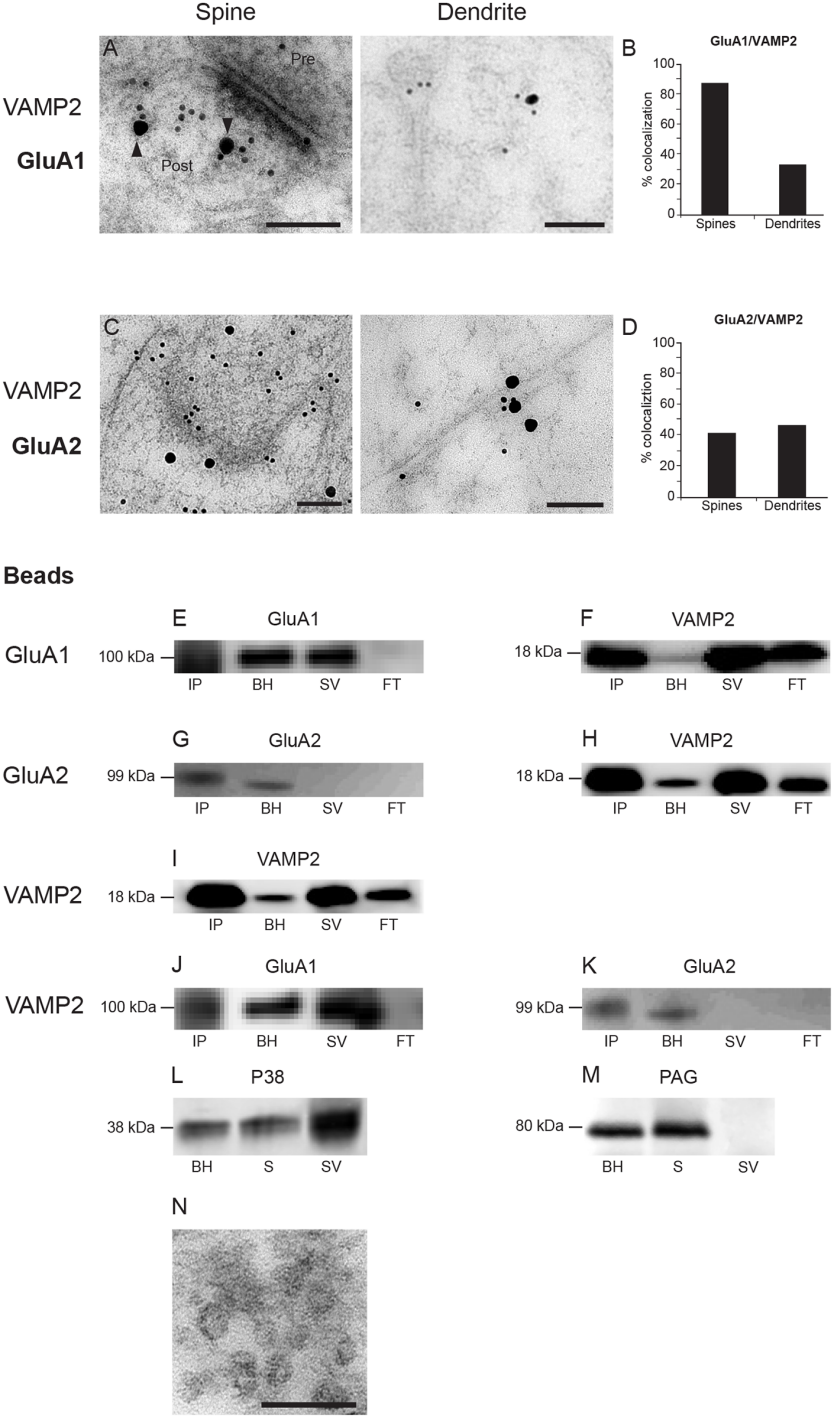
Vesicular colocalization of VAMP2 and AMPA receptor subunits. (*A*) Electron micrographs showing double immunogold labeling of VAMP2 (10 nm gold particles) and GluA1 (20 nm gold) in spine and dendrite. (B) Quantitative analysis of colocalization between VAMP2 and GluA1 in spines and dendrites. (*C*) Electron micrographs showing double immunogold labeling of VAMP2 (10 nm gold particles) and GluA2 (20 nm gold particles). (D) Quantitative analysis of colocalization between VAMP2 and GluA2 in spines and dendrites. (*E*–*K*) Immunoprecipitation (IP) of synaptic vesicles with magnetic beads coated with antibody, along with brain homogenate (BH), synaptic vesicle (SV) preparations and flow-through (FT), all subjected to gel electrophoresis and western blotting [65]. (*E*) IP with anti-GluA1, WB with anti-GluA1. (*F*) IP with anti-GluA1, WB with anti-VAMP2. (*G*) IP with anti-GluA2, WB with anti-GluA2. (H) IP with anti-GluA2, WB with anti-VAMP2. (*I*) IP with anti-VAMP2, WP with anti-VAMP2. (*J*) IP with anti-VAMP2, WB with anti-GluA1. (*K*) IP with anti-VAMP2, WB with anti-GluA2. (*L*) WB of brain homogenate fractions stained with anti-synaptophysin (P38) antibody. (*M*) WB of brain homogenate fractions stained with phosphate-activated glutaminase [39] antibody. (*N*) Electron micrograph of vesicle preparation similar to the ones used in (*E*-*M*). Scale bars: 100 nm (*A spine*, *B*), 125 nm (*A dendrite*), 100 nm (*N*).

We next wanted to explore whether the labeled postsynaptic vesicles harbored AMPA receptors, which would be expected if these vesicles contribute to insertion of AMPA receptors into the synapse, thus playing a role in synaptic plasticity. Electron microscopical postembedding immunogold double labeling, for GluA1 and VAMP2, showed that these proteins were often colocalized over vesicles in postsynaptic spines and in dendritic shafts (Fig 6A). Quantitative analysis of immunogold double labeling revealed that GluA1 and VAMP2 were clearly more often colocalized at small vesicles in dendritic spines than in dendritic shafts. Eighty-eight percent of GluA1-gold particles in spines were located close to VAMP2-gold particles, while the corresponding number for dendritic shafts was only 33% (Fig 6B), indicating that VAMP2-mediated vesicular insertion of GluA1 subunits is more common in spines than in dendrites. To further substantiate that the same vesicles could harbor both proteins, we used magnetic beads coupled to a GluA1 antibody to immunoprecipitate (IP) AMPA receptor-containing vesicles from whole rat brain synaptic vesicles. The crude brain homogenate and the synaptic vesicle preparation were positive for both VAMP2 and GluA1 (Fig 6E, 6F and 6I). Vesicles immunoprecipitated with the GluA1 beads were positive for GluA1 (Fig 6E), but also for VAMP2 (Fig 6F). Conversely, vesicles immunoprecipitated with anti-VAMP2 beads were positive for VAMP2 (Fig 6I), but also for GluA1 (Fig 6J).

We performed similar experiments with antibodies to GluA2 and VAMP2. Immunogold double labeling of excitatory synapses in hippocampus with anti-VAMP2 and anti-GluA2 more rarely showed colocalization of these proteins over vesicles in postsynaptic spines (Fig 6C). Contrary to our observations with GluA1, quantification showed only 41**%** GluA2/VAMP2 double labeling of vesicles in postsynaptic spines, which was slightly less than in dendritic shafts, where 46% of the GluA2 gold particles were colocalized with VAMP2 (Fig 6D). Vesicle immunoprecipitation experiments with GluA2 and VAMP2 antibodies gave similar results to GluA1 and VAMP2, i.e. the two proteins co-immunoprecipitated. However, a crucial difference was evident between the results with GluA1 and GluA2: While GluA1 is strongly present in synaptic vesicles (Fig 6E and 6J), GluA2 is very weak in the same fraction (Fig 6G and 6K). However, when we immunoprecipitate either VAMP2-positive or GluA2 positive vesicles from the vesicle fraction, the signal for GluA2 is clearly seen in the IP fraction (Fig 6G and 6K). Thus, contrary to GluA1, the concentration of GluA2 positive vesicles in postsynaptic spines appears to be low, but the few GluA2 positive vesicles do also contain VAMP2 (Fig 6H), as do the more numerous GluA1-positive synaptic vesicles. The levels of the two glutamate receptor subunits are much more similar in whole brain homogenate (Fig 6E and 6G). Taken together, these data indicate that GluA1 is abundant on VAMP2-positive vesicles in postsynaptic spines, relative to similar vesicles in dendrites, while GluA2-VAMP2-positive vesicles are less common in spines and more evenly distributed between dendrites and spines. Control experiments confirmed the presence of the synaptic vesicle marker synaptophysin in our vesicle preparation (Fig 6L), but they were not contaminated by nonvesicular protein, i.e. they were immunonegative for phosphate activated glutaminase [39] (Fig 6M), which is present in high concentrations in synaptic mitochondria [40]. Electron micrographs of the vesicle pellet also confirmed the purity of the preparation (Fig 6N).

### VAMP2 facilitates differential vesicle trafficking of AMPA receptor subunits

**Fig 7:**
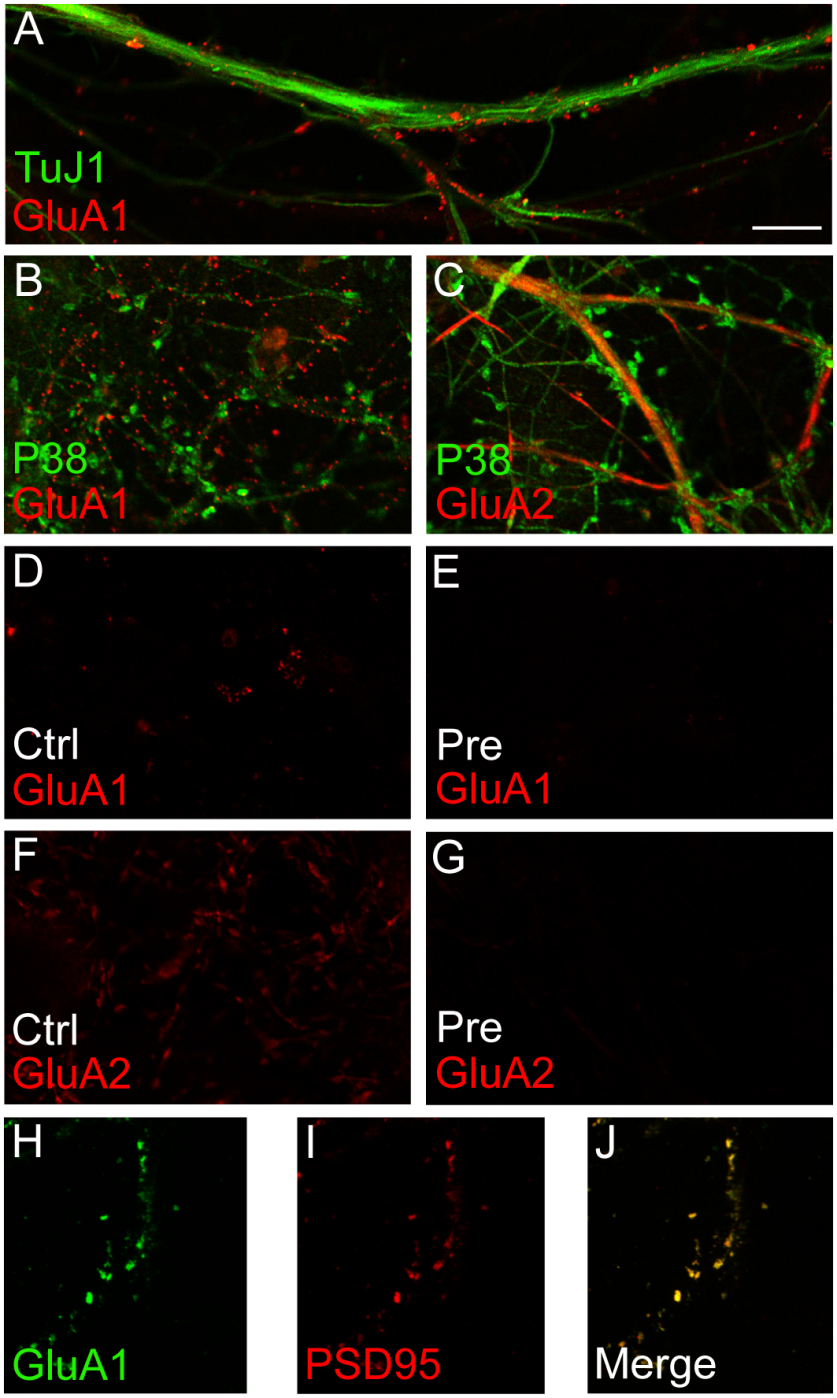
Control immunolocalization of plasma membrane AMPA receptor subunits (external epitopes) in hippocampal neuronal cultures. (*A*) Beta-tubulin (green, anti-TuJ1) labeling of dendrite, after anti-GluA1 external epitope [66] labeling and subsequent plasma membrane permeabilization. Note punctate GluA1 [66] labeling along the dendrite. (*B*) Synaptophysin (P38, green) and GluA1 [66]. (*C*) Synaptophysin (green) and GluA2 [66]. (*D*) Labeling with anti-GluA1. (*E*) Labeling with anti-GluA1, but the antibody was preincubated with the peptide antigen before staining. (*F*) Labeling with anti-GluA2. (*G*) Labeling with anti-GluA2, but the antibody was preincubated with the peptide antigen before staining. (*H*) Immunolabeling of non-permeabilized neuronal cultures with ani-GluA1 (green). (*I*) Immunolabeling with the postsynaptic marker anti-PSD95 [66] after permeabilization of neuronal cultures. (*J*) Merging of (*H*) and (*I*) showing postsynaptic labeling of GluA1. Scale bar (in A, valid for all images): 10 μm.

Having established the presence of GluA1 on VAMP2-containing vesicles in postsynaptic spines, we wanted to investigate whether VAMP2 has a functional role in exocytotic insertion of the glutamate receptor subunit into the synaptic plasma membrane. We performed immunofluorescence staining of unpermeabilized, dissociated hippocampal cultures with an antibody against the N-terminal, external epitope of GluA1, before permeabilization and subsequent immunostaining with an antibody against a somatodendritic protein, β-tubulin (TuJ1) (Fig 7A). The neurons showed punctate GluA1 labeling (Fig 7A and 7B), corresponding to synaptic sites where receptors have been inserted into the plasma membrane. Double labeling with GluA1 and PSD-95 gave almost fully overlapping punctate staining (Fig 7H–7J). Internal, cytoplasmic pools of the receptor were thus not stained. Similar labeling with anti-GluA2 (Fig 7C) did not give the typical punctate labeling pattern seen with GluA1, since the immunofluorescence was drawn out through the dendrites, not confined merely to synaptic puncta, corresponding to our previous observations at the immunogold and biochemical levels. Fig 7D–7G shows that preincubation of the two antibodies with their corresponding peptide antigens abolished staining.

**Fig 8:**
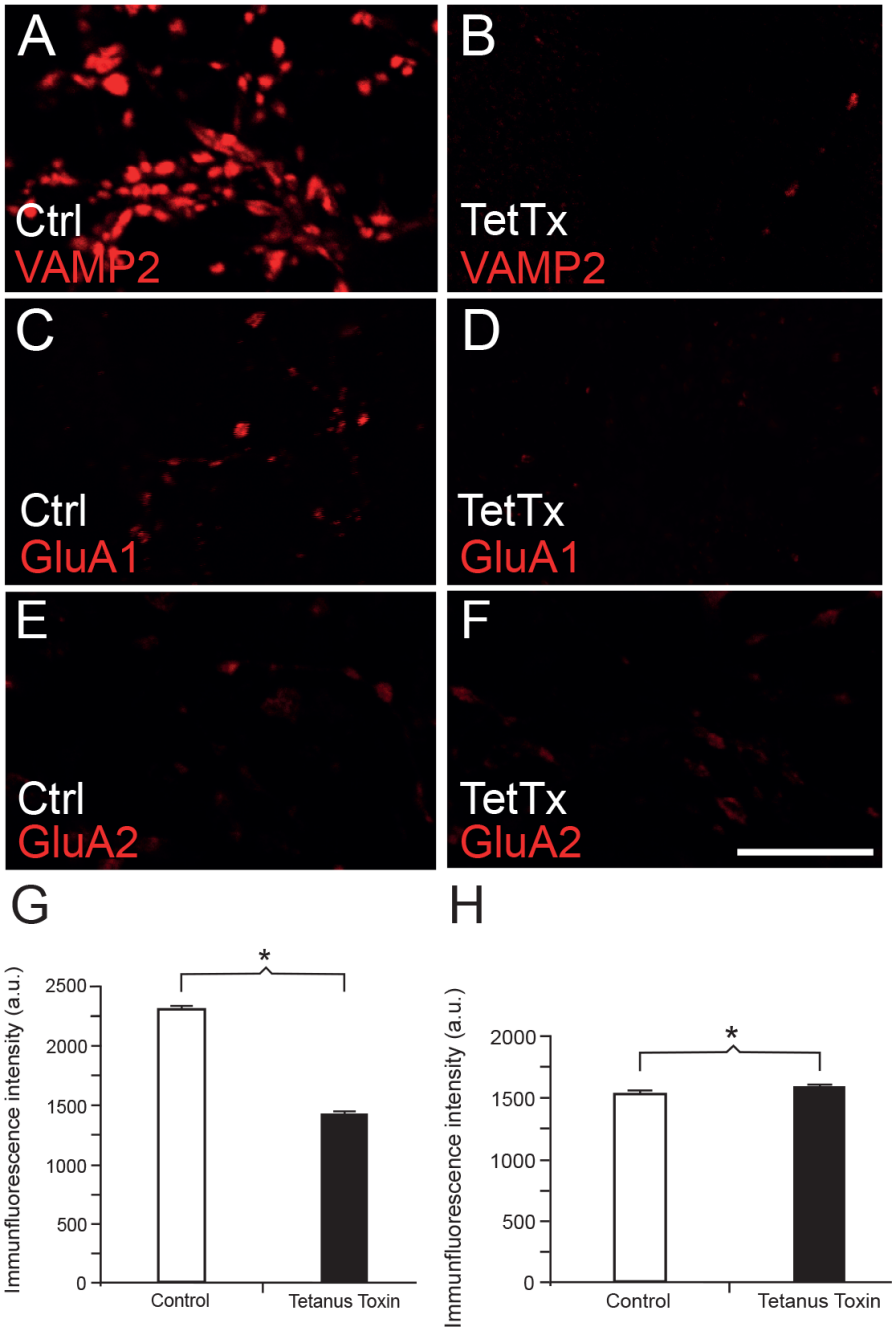
Cleavage of VAMP2 with tetanus toxin disrupts synaptic insertion of GluA1, but not GluA2, in the plasma membrane of dissociated hippocampal cultures. (*A* and *B*) VAMP2 immunofluorescence after permeabilization, in (*B*) also after tetanus toxin treatment. Note the effect of tetanus toxin, with disruption of VAMP2. (*C* and *D*) Immunofluorescence against the external epitope of GluA1, in (*D*) also after tetanus toxin treatment. (*E* and *F*) Immunofluorescence against the external epitope of GluA2, in (*F*) also after tetanus treatment. (*G*) Graphical depiction of intensity of punctate, external GluA1 labeling, showing a significant reduction of synaptic GluA1 after tetanus toxin treatment. (*H*) Graphical depiction of intensity of punctate, external GluA2 labeling, showing a slight, but significant increase of synaptic GluA2 after tetanus toxin treatment. Scale bar: 10 μm. Asterisks denote statistical significant difference (*p* < 0.001).

When cultures were treated overnight with tetanus toxin, which cleaves VAMP2 (Fig 8A and 8B), confocal images showed significantly reduced (*n* = 1000 in both groups, *p* < 0.001, independent samples *T*-test) labeling intensity of the GluA1 positive punctate structures (Fig 8C and 8D). Quantification of the maximum punctate labeling intensity showed that it was reduced to about 63% upon treatment with tetanus toxin (Fig 8G). In conclusion, our data strongly indicate that VAMP2 plays a role in insertion of the AMPA receptor subunit GluA1 into the synaptic plasma membrane.

Finally, we wanted to investigate if GluA2 is inserted by a similar VAMP2-dependent mechanism into the plasma membrane of postsynaptic spines. As noted above, the occurrence of synaptic vesicles harboring both VAMP2 and GluA2 is more evenly distributed between dendrites and spines than it is for GluA1. Tetanus toxin cleavage of VAMP2 was performed as above, but now external epitopes of GluA2 were labeled instead of GluA1 (Fig 8E and 8F). Contrary to GluA1, the intensity of GluA2 labeling of synaptic puncta after tetanus toxin treatment was slightly, but significantly, increased (*n* = 1000 in both groups, *p* < 0.001, independent samples *T*-test) (Fig 8H). Taken together, our data are compatible with a direct, regulated route for VAMP2-dependent insertion of predominantly GluA1-containing AMPA receptors into the plasma membrane in spines, both in the lateral postsynaptic membrane and directly in the synaptic plasma membrane along the PSD. Contrary to this, we find indications for a more indirect route for insertion of predominantly GluA2-containing AMPA receptors into the dendritic plasma membrane. After insertion, GluA2-receptors may possibly migrate by membrane diffusion into spines and the postsynaptic plasma membrane.

### Plasma membrane expression of AMPA receptor subunits after presynaptic silencing with Bafilomycin

**Fig 9:**
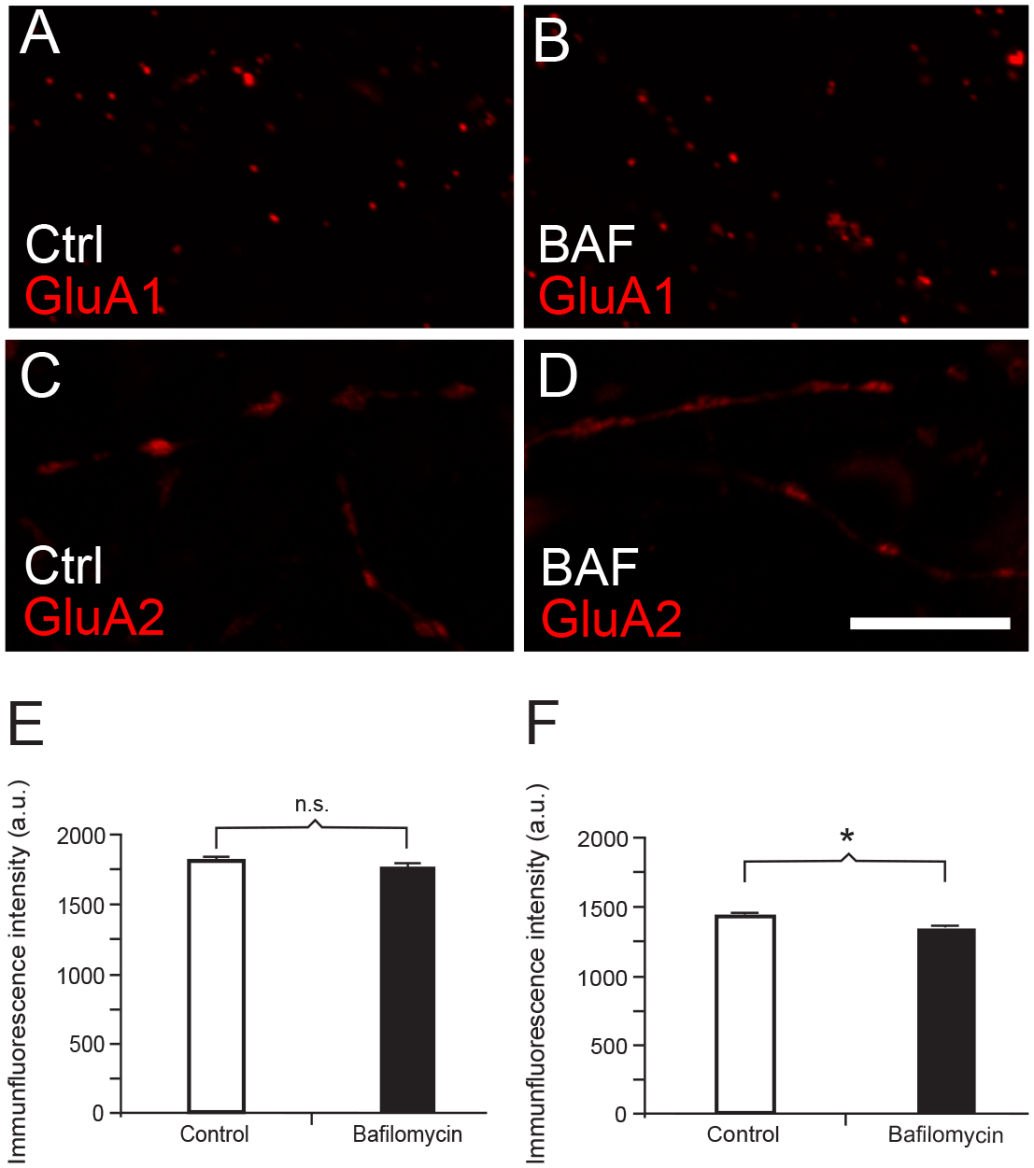
The effect on plasma membrane expression of AMPA receptor subunits in hippocampal culture after presynaptic silencing with Bafilomycin (BAF). (*A*) Control synapses immunolabeled with anti-GluA1 in non-permeabilized culture. (*B*) As in (*A*), but treated with Bafilomycin (BAF) before fixation. (*C*) Control synapses immunolabeled with anti-GluA2 in non-permeabilized culture. (*D*) As in (*C*), but treated with Bafilomycin (BAF) before fixation. (*E*) Quantification of difference in maximal punctate GluA1 labeling intensities between control and Bafilomycin-treated cultures. There is no significant difference. (*F*) Quantification of difference in maximal punctate GluA2 labeling intensities between control and Bafilomycin-treated cultures. There is a slight, but significant decrease in labeling intensities after Bafilomycin treatment. Asterisk denotes statistical significance (*p* < 0.001). Scale bar: 10 μm.

Treatment with tetanus toxin, however, will also inactivate VAMP2 in the presynaptic terminal, and as a result reduce transmitter release. Blocking of vesicular refilling of glutamate with Bafilomycin A1 was performed in cultures not treated with toxin to control for the effect of presynaptic inactivity. Bafilomycin is a potent blocker of vacuolar ATPases [41] and inhibits cross-membrane vesicular transport of neurotransmitter by inhibiting acidification of the vesicles. We have previously shown that Bafilomycin A1 significantly decreases synaptic vesicular release of glutamate in these hippocampal cultures [42]. Treatment of neuronal cultures with Bafilomycin gave no significant reduction (*n* = 500 in both groups, *p* = 0.195, Mann-Whitney *U* test) in maximum punctate immunofluorescence intensity of GluA1 (Fig 9A, 9B and 9E). Thus, in the present setup, presynaptic inactivity does not affect the plasma membrane expression of GluA1. Bafilomycin inhibition of presynaptic release gave a slight, but significant decrease (*n* = 500, *p* < 0.001, Mann-Whitney *U* test) of surface GluA2 expression (Fig 9C, 9D and 9F).

## Discussion

In spite of a vast amount of research on the trafficking of glutamate receptors, we still do not know the details of the molecular mechanisms for delivery of glutamate receptors to the postsynaptic plasma membrane. The present investigation provides information on important aspects of these mechanisms. First, we demonstrate the presence of small postsynaptic vesicles and characterize their size and intrasynaptic localization. Second, we show that the SNARE protein VAMP2 is associated with many of these vesicles, strongly substantiating the notion that SNARE proteins regulate postsynaptic vesicle trafficking. Third, a significant proportion of these postsynaptic vesicles harbor GluA1-containing AMPA receptors, and our results strongly indicate that VAMP2 facilitates the insertion of these receptors into the postsynaptic plasma membrane. Finally, our data indicate differential trafficking of GluA1 and GluA2 into the spine plasma membrane, with GluA1 mostly taking a direct vesicular route from the spine cytoplasm, while GluA2 may take an indirect route, being inserted into dendritic plasma membrane before migrating to the synapse.

Our results rely heavily on antibody specificity and selectivity. We have controlled for these factors in the different experiments, but acknowledge that, e.g. different sensitivities between GluA1 and GluA2 antibodies may possibly influence our findings. Many of the VAMP2-labeled vesicles in our material show the immunogold particle close to the center of the vesicle, inside the electron-lucent vesicle over the interior. This does not imply that the epitope is actually localized in the vesicular lumen. Because the epitope is bound to a complex of primary and secondary antibodies coupled to a gold particle, the center of an immunogold particle can be located up to 21 nm from the epitope. In the case of synaptic vesicle proteins like VAMP2, a single immunogold particle is often attached by more than one primary antibody around the circumference of the vesicle membrane, and it will thus be seen over the interior of the vesicle, as is evident for other vesicle proteins as well [33, 43, 44].

Past research has provided indirect evidence for a vesicular pathway of local insertion of AMPA receptors into the spine plasma membrane [6, 10, 11, 23–25]. The putative vesicles, however, have never been demonstrated. When the SNARE complex and its function in presynaptic vesicle exocytosis was first characterized [18], the existence of presynaptic vesicles in the brain [45] and their exocytosis of transmitter [46, 47] had already been demonstrated. With postsynaptic spines, the situation is different. We know that glutamate receptors migrate from cytoplasmic stores to the postsynaptic plasma membrane [48]; indirect evidence, i.a. the involvement of SNARE proteins [9, 11, 25, 48], support the notion that the receptors move to the plasma membrane by exocytosis, but the organelles themselves have actually not been shown. Thus, our ultrastructural demonstration of such vesicles within the postsynaptic cytoplasm (Fig 3) fills a gap in current knowledge about postsynaptic spine biology.

The proteins mediating the direct transfer from a postsynaptic spine cytoplasmic pool to a plasma membrane pool are only just beginning to be determined. A strong indication of SNARE complex involvement was provided by Kennedy and colleagues when they showed that syntaxin-4 was involved with the exocytosis of AMPA receptors in dendritic spines [10]. Later, Jurado et al. [11] showed that the SNARE proteins syntaxin-3 and SNAP-47 are required for regulated AMPA receptor exocytosis during LTP, but not for constitutive basal AMPA receptor exocytosis. The same study showed that VAMP2 contributes to both regulated and constitutive AMPA receptor exocytosis. Similarly, Araki and colleagues [49] showed that VAMP2 is involved with insertion of GluA2-containing vesicles, but they did not differentiate between dendritic shafts or spines.

Our results with electron microscopy, western blotting and co-immunoprecipitation take this line of research further, showing that postsynaptic vesicles may harbor both VAMP2 and GluA1 (Figs 3 and 6). Furthermore, by disrupting VAMP2 with tetanus toxin, we find evidence that this SNARE protein is instrumental for exocytosis of GluA1-containing receptors in the postsynaptic membrane. Tetanus toxin treatment, however, gave a small, but significant, increase in synaptic concentrations of GluA2. This observation indicates that decreasing concentrations of synaptic GluA1-containing receptors may be replaced by lateral diffusion of GluA2-containing receptors from a dendritic plasma membrane pool. As a control for silencing of inactive synapses, i.e. when we treat them with tetanus toxin, reducing their glutamate release, we used Bafilomycin (Fig 9). Bafilomycin inhibits filling of presynaptic vesicles with glutamate so that little glutamate is released from the axon terminals. This treatment did not result in a reduction in surface expression of GluA1, as did the toxin. On the other hand, other studies have shown that reducing transmitter release may cause an increase in surface expression of AMPA receptors in culture [50, 51]. This scaling effect, however, typically takes two or three days to appear, much longer than our overnight treatment with Bafilomycin.

There are two interesting aspects of our observations that should be discussed: First, VAMP2- and GluA1-labeled vesicles in many cases touched the spine plasma membrane, both in perisynaptic areas as well as at the PSD, in addition to the many labeled vesicles within the spine cytoplasm (Fig 3). This observation supports a direct route of insertion close to, or even directly into the plasma membrane corresponding to the PSD, as indicated by some investigations [10, 52, 53], but not by others [54, 55]. Secondly, we find indications that GluA1- and GluA2-containing receptors may be differentially inserted into the plasma membrane. First of all, they are affected differently by disrupting VAMP2 with tetanus toxin. Furthermore, blots from synaptosomal vesicles are only very weakly GluA2-positive, while they are strong for GluA1, but this difference is not evident in blots from whole brain homogenate (Fig 6E and 6G). This is supported by the electron microscopical observations that vesicles co-harboring VAMP2 and GluA1 are abundant in spine cytoplasm compared to dendrites, while similar vesicles with VAMP2 and GluA2 are almost evenly distributed between dendritic shafts and spines (Fig 6A–6D). Also, the confocal images of hippocampal, cultured neurons are in line with this, showing GluA1 labeling in distinct puncta and GluA2 labeling along segments of dendrites (Fig 7). While many researchers have favored insertion of fully assembled AMPA receptors into the plasma membrane, e.g. in dendrites, our findings of differential localization and trafficking of GluA1 and GluA2 indicate that exocytosis of AMPA receptor subunits may occur in both spine and dendritic plasma membrane, respectively. Our results, then, are also compatible with increasing evidence for differential trafficking of GluA1- and GluA2-containing receptors [15, 16].

Our distinction between GluA1- and GluA2-containing AMPA receptors warrants some discussion, as many AMPA receptors are believed to be heteromeric, containing both of these subunits. Differential trafficking and insertion of AMPA receptors are of crucial importance to synaptic plasticity and adaptation to different pathological or physiological conditions. In the synapse, the GluA1/GluA2 heteromeric receptor is one of the major subunit compositions [56]. Approximately 80% of synaptic and > 95% of somatic extrasynaptic receptors are believed to be GluA1/A2 heteromers. However, in tissue extracts from the CA1 and CA2 areas of the hippocampus, around 8% of the AMPA receptors were shown to be GluA1 homomers, with a very small population of GluA1/GluA3 heteromers [57]. Furthermore, the molecular composition of synaptic AMPA receptors can change in response to activity [58–60]. In summary, there is a need for elucidating the mechanisms for regulating the subunit composition of synaptic AMPA receptors. Interpreted on the basis of previously published studies, our results indicate that VAMP2 may facilitate vesicular insertion of GluA1-containing homomeric or GluA2-lacking AMPA receptors into spines, while VAMP2 also could be involved with insertion of GluA2-containing AMPA receptors into the dendritic plasma membrane before they may migrate laterally to the spines.

There is also evidence that AMPA receptors may be stored in recycling endosomes [61]. In our synapses, endosomic structures could sometimes be seen in spines, but they were not very common. In synapses where recycling endosomes are important stores of AMPA receptors, small vesicles could play a role in trafficking receptors to the plasma membrane and back. Interestingly, work with another set of vesicle or endosomal trafficking proteins, Rab proteins, also support trafficking of GluA1-containing endosomes into postsynaptic spines before insertion into the postsynaptic plasma membrane [62]. Specifically, the authors found that Rab11-dependent endosomes traffic GluA1-containing AMPA receptors from dendritic shafts into spines. Subsequently, an additional endosomal trafficking step, controlled by Rab8, drives receptor insertion into the synaptic membrane. In light of our own findings, which indicate separate insertion routes for GluA1 homomeric/GluA2-lacking receptors and GluA2-containing receptors, it would have been very interesting to know whether other Rab proteins control GluA2-containing AMPA receptors. Further work will show if our VAMP2/GluA1-positive vesicles also harbor Rab8 or Rab11.

The two VAMP genes abundantly expressed in the brain are VAMP1 and VAMP2. The protein detected in the hippocampal postsynaptic spines in the present study is very likely VAMP2, as VAMP1 is expressed mainly in the spinal cord and only in low concentrations in the hippocampus [27], and tetanus toxin only cleaves VAMP2, not VAMP1 [63]. Furthermore, tetanus toxin does not cleave VAMP2 that is already assembled in a SNARE complex [38]. Thus, even after toxin treatment, there will be some insertion of GluA1 receptor subunits from already docked vesicles.

In conclusion, our data show the presence of small, VAMP2-positive vesicles in postsynaptic hippocampal spines, colocalized with GluA1. Our results support a direct route of GluA1-containing AMPA receptor insertion into the spine plasma membrane, while GluA2-containing AMPA receptors are predominantly inserted into the dendritic plasma membrane, supporting a lateral migration into the spine. In both cases, the insertion occurs by VAMP2-mediated exocytosis of receptor-containing small vesicles. VAMP2 is well-known for its facilitation of presynaptic vesicle exocytosis. But the two compartments, the presynaptic terminal and the postsynaptic spine, are isolated by two plasma membranes from each other, so the same SNARE proteins, in this case VAMP2, may easily function in the exocytosis of different sets of vesicles, in different locations. As pointed out by Jurado et al. [11], however, different sets of postsynaptic target SNAREs (syntaxins) may be involved with different types of exocytosis, i.e. with regulated or constitutive exocytosis, respectively.
